# Psychometric properties of the Persian version of emotions and attitudes towards MRI” (MRI-EMA) 

**DOI:** 10.22088/cjim.14.3.449

**Published:** 2023

**Authors:** Abdorreza Naser Moghadasi, Sara Hamtaei Gashti, Mohammad Ali Sahraian, Mahsa Ghajarzadeh

**Affiliations:** 1 Multiple Sclerosis Research Center, Neuroscience Institute, Tehran University of Medical Sciences, Tehran, Iran; 2 Universal Council of Epidemiology (UCE), Universal Scientific Education and Research Network (USERN), Tehran University of Medical Sciences, Tehran, Iran

**Keywords:** Multiple sclerosis, Magnetic resonance imaging, Anxiety.

## Abstract

**Background:**

Patients with multiple sclerosis (MS) should have magnetic resonance imaging evaluation regularly. They will experience anxiety before this examination. We conducted this study to evaluate the validity and reliability of emotions and attitudes towards MRI” (MRI-EMA).

**Methods:**

One hundred-nine patients with MS were asked to fill the valid and reliable Persian version of Beck Anxiety Inventory (BAI), and MRI-EMA, questionnaires. Two weeks later, twenty cases were asked to fill the questionnaire again to assess reliability. The intra-class correlation coefficient (ICC) and Cronbach's alpha analysis were used. The correlation coefficient between BAI and MRI-EMA was calculated. Five neurologists assessed content validity by content validity ratio (CVR) and content validity index (CVI).

**Results:**

The mean age was 37.2±1.2 years and 77% were females. CVI and CVR for all questions were 100%. The correlation coefficient between BAI and MRI-EMA was r=0.1, P=0.1 and only fear of MRI subscale was significantly correlated with BAI. The ICC of all questions was between 0.79 and 0.98.

**Conclusion:**

Patients with MS have to be routinely screened with MRI which provides anxiety for them. Considering MRI related anxiety is crucial for these cases. The Persian version of the MRI-EMA questionnaire is a valid and reliable instrument for measuring MRI related anxiety in patients with multiple sclerosis.

Multiple sclerosis (MS) is a disabling neurological disease of the central nervous system (CNS) affecting women more than men (1). After the diagnosis, they have to be monitored by clinical examination and magnetic resonance imaging (MRI) to determine the disease status and efficacy of medications. There is a challenge regarding the association between imaging findings and clinical manifestations in MS cases and the number of lesions does not match the clinical findings (2) but most clinicians rely on imaging findings when they want to assess the efficacy of administered medications (3). On the other hand, a systematic review showed that MRI is a source of anxiety for MS patients (4). In a study which was conducted by Chalfant et al., 15% of enrolled MS patients experienced posttraumatic stress disorder (PTSD) for diagnostic information (5). 

Engels et al. developed a questionnaire assessing feelings towards magnetic resonance imaging (MRI) in people with multiple sclerosis (MRI-EMA) and found that receiving MRI results caused anxiety in cases with MS (6). The number of MS patients is increasing rapidly in Iran and MRI is a routine evaluation method for them (7). 

So, we designed this study to evaluate the validity and reliability of the Persian version of emotions towards magnetic resonance imaging in Iranian MS patients. 

## Methods

We conducted this cross-sectional study in the MS clinic of Sina Hospital (affiliated hospital of Tehran University of Medical Sciences) between March and September 2020. Inclusion criteria were: MS diagnosis based on McDonald’s revised criteria (8), and age older than 18. Exclusion criteria: Definite cognitive impairment, cases with the diagnosis of generalized anxiety disorder. 

We asked the patients to fill informed consent form. The study protocol was approved by the Ethics Committee of the Neuroscience Institute of TUMS (IR.TUMS.NI.REC.1398.038). 

We asked all patients to fill valid and reliable Persian version of Beck Anxiety Inventory (BAI). BAI has 21 questions, each rating from 0 to 3. The total score can range between 0-63 (9). MRI-EMA has 10 questions, each could be answered through a four-scale rating (1= I don’t agree at all, 4= I completely agree) including four factors: fear of MRI scan, fear 62 of MRI results, feeling of control over the disease and feeling of competence. Total score is the sum, of all questions. Using the forwardbackwards translation method, a bilingual medical researcher translated the (MRI-EMA) questionnaire into the Persian. Then, the Persian version was re-translated into English. This section was done by alternative bilingual medical researcher. Then, a neurologist compared the two versions. Content validity was checked by five neurologists and content validity ratio (CVR) and content validity index (CVI) were calculated. To assess construct validity, the correlation coefficient between BAI and MRI-EMA was calculated. Two weeks after the initial assessment, we asked twenty cases to fill the questionnaire again to assess reliability. 

The intraclass correlation coefficient (ICC) was used for repeatability, and ICC coefficients more than 0.70 were considered acceptable. We used SPSS software Version 22 (SPSS Inc., Chicago, IL, USA) to analyze the data. Data are presented as mean ± SD for continuous or frequencies for categorical variables. Cronbach’s alpha was calculated for subscales to assess the internal consistency reliability. Cronbach’s alpha coefficient ≥0.70 was considered as excellent reliability. If p- value was less than 0.05, we considered it as significant.

## Results

We enrolled 109 patients. The mean age was 37.9±1.2 years and 77% were females. Data of enrolled cases are shown in table 1. CVI and CVR for all questions were 100%.The mean scores of BAI and MRI-EMA were 15.4±10.9 and 24.4±5.3. The mean scores of MRI-EMA subscales and the correlation coefficient between BAI and MRI-EMA are shown in table 2. Cronbach’s alphas of subscales were between 0.73-0.85 (table 3). The ICC of all questions was between 0.79 and 0.98 (table 4). The mean total MRI-EMA and its subscales scores were not significantly different between two groups based on disease duration (≤ 5 years and more than 5 years).

**Table 1:** Basic characteristics of enrolled cases

**Result**	**Variable**
37.2±1.9	**Age (mean ± SD) (year)**
84(77.1%) 25(22.9%)	**Sex** **Female** **Male**
74(67.9%) 35(32.1%)	**Marital status** **Married** **Single**
47(43.1%) 62(56.9%)	**Work status** **Employed** **Unemployed**
8.5±4.6	**Disease duration (mean ± SD) (year)**
2.1±3.8	**EDSS(mean ± SD)**

EDSS: Expanded Disability Status Scale

**Table 2:** The mean scores of subscales and their correlation coefficient with BAI

**P-value**	**Correlation coefficient**	**Mean score**	**Subscale**
0.01	0.24	3.7±1.7	**Fear of MRI scan**
0.17	0.13	6.5±2.6	**MRI results**
0.9	0.09-	8.4±2	
0.4	0.08	5.5±1.5	**Feeling of competence**

MRI: Magnetic Resonance Imaging

**Table 3:** Cronbach’s alpha of the items

**Cronbach’s alpha**	**Items**
0.73	**Fear of MRI scan**
0.85	**MRI results**
0.77	**Feeling of control**
0.8	**Feeling of competence**

MRI: Magnetic Resonance Imaging

**Table 4:** The ICC of the questions

**Cronbach’s alpha**	**Items**
0.94	
0.91	
0.91	
0.82	
0.9	
0.91	
0.79 0.92	
0.98	
0.91	

MRI: Magnetic Resonance Imaging

**Table 5:** Comparison of items between patients with < or > 5 years of disease duration

**P-value**		**≤ 5 years**	
0.7	3.7±1.8	3.5±1.6	**Fear of MRI scan**
0.7	6.3±2.6	6.1±2.6	**MRI results**
0.6	8.6±2.1	8.3±2.1	**Feeling of control**
0.4	5.6±1.5	5.4±1.7	**Feeling of competence**
0.8	24.3±5.6	24.1±5.4	**Total**

MRI: Magnetic Resonance Imaging

## Discussion

We assessed the validity and reliability of the Persian version of MRI-EMA. This questionnaire was developed by Engels et al. to assess emotions toward MRI in patients with MS.The ICC values were high (ranging between 0.79-0.98) showing valuable and significant testretest reliability of the questionnaire. We also found that Cronbach’s alpha values of subscales were between 0.73-0.85 showing high internal consistency of each item. The mean score of fear of MRI was higher in our study, 3.7 vs the mean score in Engles et al.’s study which was 2.5 (6). The mean score of all other three items was also lower in Engles et al.’s study than ours (MRI results: 2.5 vs 6.5, control: 2.7 vs 8.5, and competence 2.6 vs 5.5). This could show that Iranian MS patients suffer more from MRI related anxiety.

As the results show, there was a significant positive correlation between BAI and fear of MRI subscale while there was no significant correlation between BAI and other subscales. MRI is a choice of diagnostic and follow-up modality in MS patients which is used frequently, but little is known about the perception of Iranian patients regarding this modality. Our results show that MS patients have anxiety regarding MRI scans and also the results of the scan. This could be due to detection of the silent disease activity and accidental detection of new plaques in MS patients which could change the management of the disease.In our study, there was no significant difference between two groups of the patients (different disease durations).

It was suggested that by growing MRI experience during the time, the anxiety experience of the patients decrease but we did not detect this difference. In Engles et al.’s study, the means score of MRI results related anxiety score was significantly higher and competence score was significantly lower in cases with disease duration less than 5 years (6). This could show that our patients suffer from MRI-related anxiety independent of disease duration and previous experiences. As our results show there was a significant positive correlation between BAI and MRI scan which confirms other findings. A previous study showed that education regarding MRI will decrease anxiety level and increase competence(10). We lack this education in Iran and we have to develop a new program to educate MS patients regarding MRI to decrease anxiety level.

This study had some strengths. First, it is done for the first time in Iran. Second, we validated the MRI-EMA questionnaire which could be used in later studies. This study had restrictions. It was conducted in one tertiary center. Second, we did not assess the relationship between disease status and level of anxiety. Larger, multi-centric studies are recommended. Conclusion: Patients with MS have to be routinely screened with MRI which provides anxiety for them. Considering MRI-related anxiety is crucial for these cases. The Persian version of the MRI-EMA questionnaire is a valid and reliable instrument for measuring MRI-related anxiety in patients with multiple sclerosis.
